# Supplementary material for: A Likelihood Approach for Real-Time Calibration of Stochastic Compartmental Epidemic Models
Source: PLoS Comput Biol. 2017 Jan 17;13(1):e1005257. doi: 10.1371/journal.pcbi.1005257 (PMC5240920; doi:10.1371/journal.pcbi.1005257)
Supplement: S1 File — (TAR.GZ) [file pcbi.1005257.s014.tar.gz › HSPH_Online-SI-Revision/EnKF07/n1-mild/n1-mild_PF_EnKF-summery.pdf]

peak -8	R0	$1/\theta_2$	inf	inflog	newly	newlylog	Reff	AttackRate
mean	91.8	57.1	1295.2	0.9	2334.6	1.	59.5	272.6
median	90.9	53.5	933.9	0.9	1247.5	0.9	64.1	283.3
Quantile .1	72.1	38.7	156.1	0.4	278.6	0.4	39.2	168.3
Quantile .9	106.6	74.5	2425.1	1.3	5757.7	1.5	70.8	353.4
29	31	3						
length	50							

peak -4	R0	$1/\theta_2$	inf	inflog	newly	newlylog	Reff	AttackRate
mean	100.7	54.3	283.6	0.5	514.3	0.5	63.7	317.5
median	98.8	54.6	169.1	0.4	354.	0.5	65.3	298.6
Quantile .1	83.	39.3	92.7	0.3	154.7	0.4	56.	234.4
Quantile .9	123.	70.1	587.9	0.7	892.9	0.8	70.	411.2
21	27	0						
length	50							

peak	R0	$1/\theta_2$	inf	inflog	newly	newlylog	Reff	AttackRate
mean	105.3	54.9	117.8	0.4	235.3	0.5	62.5	319.8
median	105.7	56.8	112.9	0.4	225.1	0.4	62.6	305.5
Quantile .1	82.7	38.3	79.1	0.3	118.4	0.4	55.3	253.8
Quantile .9	124.2	70.8	158.9	0.5	391.5	0.5	68.9	390.6
13	30	0						
length	50							

peak +4	R0	$1/\theta_2$	inf	inflog	newly	newlylog	Reff	AttackRate
mean	106.1	55.6	1038.2	0.5	1558.5	0.5	63.3	306.9
median	107.	53.8	177.2	0.4	305.8	0.5	63.6	290.
Quantile .1	82.5	32.1	67.6	0.3	107.3	0.3	56.3	244.4
Quantile .9	125.6	75.7	387.5	0.6	843.2	0.6	69.5	391.8
8	25	0						
length	50							

peak +8	R0	$1/\theta_2$	inf	inflog	newly	newlylog	Reff	AttackRate
mean	106.1	56.4	5601.8	0.7	8231.1	0.9	62.7	302.3
median	107.9	55.7	396.	0.5	829.4	0.7	62.7	284.7
Quantile .1	82.	34.8	101.2	0.3	309.9	0.5	54.	230.5
Quantile .9	120.7	74.2	4017.2	1.3	9505.	1.5	69.9	389.4
15	28	0						
length	50							
